# Eltrombopag Dose Adjustment During Infection-Induced Thrombocytosis in a Patient With Chronic Idiopathic Thrombocytopenic Purpura

**DOI:** 10.7759/cureus.14166

**Published:** 2021-03-29

**Authors:** Mohanad Ahmed, Elabbass Abdelmahmuod, Elrazi A Ali, Mohamed A Yassin

**Affiliations:** 1 Internal Medicine, Hamad Medical Corporation, Doha, QAT; 2 Hematology and Oncology, Hamad General Hospital, Doha, QAT

**Keywords:** itp, infection induced thrombocytosis, eltrombopag

## Abstract

Idiopathic thrombocytopenic purpura (ITP) is an immune disorder in which antibodies attack platelets, leading to platelet destruction and increased bleeding risk. Standard treatment is to maintain a platelet count sufficient to mitigate the bleeding risk. First-line therapies include steroids and IV immunoglobulins, and second-line therapy includes thrombopoietin receptor agonists like eltrombopag in combination with other medications (e.g., rituximab) to reduce immune attack. Eltrombopag is a nonpeptide oral thrombopoietin (TPO)-receptor agonist that increases platelet counts by binding to and activating the human TPO receptor. While using eltrombopag, the target platelet count range is usually between 50,000/mm³ and 200,000/mm³, so the dose should be adjusted accordingly. However, this dose adjustment is based on platelet count increments in response to eltrombopag administration. Adjusting the dose when the platelet count is elevated due to a different factor can be challenging. Data are not yet available on whether stopping the treatment or reducing the dose will harm the patient or result in an acute drop in platelet count and increased bleeding. We present the case of a 60-year-old woman with ITP on a stable eltrombopag regimen who completed an eltrombopag-free period after developing infection-induced thrombocytosis.

## Introduction

Eltrombopag is a small molecule, nonpeptide thrombopoietin-receptor agonist administered orally. It is approved for the treatment of thrombocytopenia to increase platelet count and protect patients from bleeding. It is usually very well tolerated orally and has an excellent safety profile with an overall positive response and improvement in around 60%-80% patients [1]. Eltrombopag response is tracked via multiple measurements of platelets count. Then, the dose is adjusted to achieve adequate platelet count to avoid both thrombocytopenia and thrombocytosis. Eltrombopag can be used as first-line or second-line therapy, alone or in combination with other medications for idiopathic thrombocytopenic purpura (ITP) [2].

In clinical practice, the term ‘thrombocytosis’ refers to platelet counts above 450,000/mm³ [3]. It can be primary or secondary to other conditions because platelets serve as an acute phase reactant.

Eltrombopag stimulates the bone marrow to produce platelets, and the dosage is adjusted based on the patient’s response to the treatment [4]. In clinical practice, some patients already on eltrombopag may exhibit an increase in platelet counts due to conditions other than the action of eltrombopag. Such conditions present challenge to physicians in making appropriate dose adjustments, especially without knowing the effect of eltrombopag withdrawal on overall safety, including bleeding risk, especially for stable patients on a fixed dose for a long time. We present our experience in adjusting the eltrombopag dose for a female patient during secondary reactive thrombocytosis due to infection.

## Case presentation

A 60-year-old woman was diagnosed with ITP in October 2018. At that time, her platelet count was 61,000/mm³. Her ITP diagnosis was made by exclusion, and she was started on steroid therapy. One week later, her platelet count decreased from 61,000/mm³ to 33,000/mm³ while on the steroid treatment. The patient was started on eltrombopag as a second-line option at a dose of 25 mg daily. At her two-week follow-up evaluation, the platelet count improved to 137,000/mm³.

Approximately six weeks later, her platelet count dropped to 40,000/mm³. Her eltrombopag dose was increased to 50 mg daily, and she had good responses on her subsequent follow-up appointments, where her platelet counts improved to 121,000/mm² within one month and to 203,000/mm³ two weeks later.

The patient maintained adequate platelet counts throughout 2019 on eltrombopag 50 mg daily. In April 2020, she developed left foot pain, redness, and swelling. She presented to the hospital, and was noted to have evidence of infection and high levels of inflammatory markers. Following a diagnosis of cellulitis, the patient was started on an oral antibiotic (amoxicillin with clavulanate) and pain medications. At that time, her platelet count increased to 536,000/mm³. She was on a stable eltrombopag dose, and her platelet count was within the reference range for an extended period before she developed the infection. We assumed that her acute increase in platelet count was secondary to an infection, as platelets are acute phase reactants. Her eltrombopag treatment was stopped to reduce her risk of developing thrombosis and monitor her closely.

**Figure 1:**
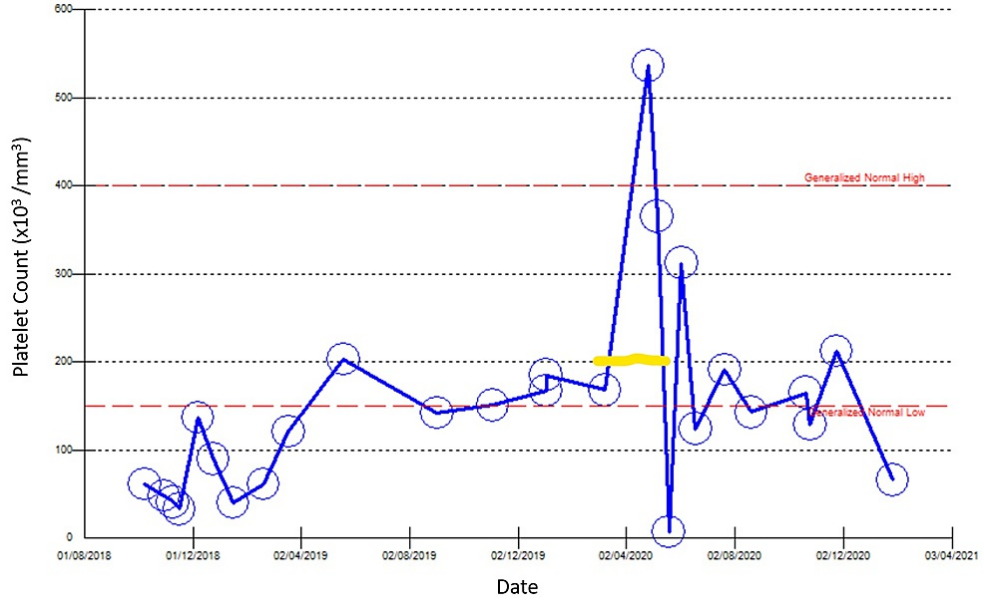
Platelet counts before, during, and after the eltrombopag-free period (the yellow line represent eltrombopag free period).

As she received treatment for cellulitis, her platelet count was maintained between 150,000/mm³ and 200,000/mm³ over the following two weeks after stopping eltrombopag without a significant drop in platelet count or bleeding. Two weeks later, the patient finished her antibiotic treatment, and her clinical condition improved. Her platelet count was 100.000/mm³. Eltrombopag was restarted, and her platelet count remained stable. Her final platelet count was 143,000/mm³ while remaining on eltrombopag 50 mg daily (Figure 1).

## Discussion

Eltrombopag is a relatively new oral medication that stimulates the bone marrow to produce platelets. Eltrombopag was used to correct thrombocytopenia due to various conditions such as ITP, aplastic anemia, and chronic hepatitis C infection-associated thrombocytopenia [5-6]. Eltrombopag may help to control the condition but will not cure it.

Eltrombopag is usually started with a low dose at 12.5 mg or 25 mg daily. The dose is gradually increased to maintain a minimum platelet count of >50,000/mm³ to prevent bleeding. The dose of eltrombopag is adjusted as follows [4]: if the patient’s platelets count <50,000/mm³, the dose should be increased gradually to reach a minimum count of 50,000/mm³ (the maximum dose is 75 mg daily). If the patient’s platelet count is between 50,000/mm² and 200,000/mm³, the dose should be maintained with no change. If the patient’s platelet count is between 200.000/mm³ and 400,000/mm³, the dose should be reduced by 25 mg daily (if the patient is taking 25 mg, the dose should be reduced to 12.5 mg daily). Finally, if the patient’s platelet count is >400,000/mm³, withhold the patient’s dose, monitor platelet count twice weekly, and when the platelet count <150,000/mm3, resume the patient’s daily dose but reduce it by 25 mg (if the patient is taking 25 mg once daily, resume with 12.5 mg once daily). These modifications are based on platelet count increments directly resulting from eltrombopag use. 

We followed the guidelines by starting our patient on an initial dose of 25 mg daily, and then we increased her dose to 50 mg daily to reach a stable platelet count above 50,000/mm³. Her condition gradually improved, and she was doing very well with a stable platelet count and no bleeding for more than one year. When she developed cellulitis, her platelet count jumped to 536,000/mm³ and presented a challenging clinical situation. No recommendations were present in the literature on adjusting her dose of eltrombopag in reactive thrombocytosis.

We chose to stop eltrombopag to avoid thrombus formation risk given that her platelet count could drop rapidly, and bleeding may occur due to underlying ITP. Fortunately, the patient maintained her platelet count above 50,000/mm³ throughout the treatment of her cellulitis. Neither an acute drop in platelet count nor bleeding occurred, and infection-induced reactive thrombocytosis was enough to prevent bleeding during the eltrombopag-free period.

## Conclusions

Eltrombopag can be safely stopped during infection-induced thrombocytosis without an acute drop in platelet count or increased bleeding risk. However, more studies are needed to reproduce the same finding. Physicians should always consider individual clinical situations and medical history when adjusting therapies.
